# Restless Legs Syndrome Presenting as an Acute Exacerbation of Multiple Sclerosis

**DOI:** 10.1155/2011/872948

**Published:** 2011-07-05

**Authors:** James H. Bernheimer

**Affiliations:** Mercy Medical Center, Baltimore, MD 21202, USA

## Abstract

Restless legs syndrome is common in patients with multiple sclerosis but has not been reported as occurring due to an acute, inflammatory, demyelinating attack. Restless legs syndrome is known to be related to low brain iron levels. Multiple sclerosis has been associated with the abnormal accumulation of iron in the chronic, progressive phase of axonal degeneration. Iron deficiency may play a role in demyelination. This suggests that restless legs syndrome may be caused by the inflammatory, demyelinating component of multiple sclerosis rather than axonal degeneration. The author presents a case of self-limited restless legs syndrome occurring as an acute attack of multiple sclerosis, supporting the notion that inflammatory demyelination is the underlying pathophysiology of restless legs syndrome in multiple sclerosis.

## 1. Case Report

The patient was a 25-year-old, right-handed woman who presented two months after the sudden onset of lower extremity paresthesias and sensory loss that extended into the abdomen and lower chest and one episode of urinary urgency and subsequent incontinence. Her examination was notable for brisk lower extremity deep tendon reflexes but no other abnormalities. MRI of the cervical and thoracic spine revealed both cervical and midthoracic plaques consistent with demyelination. A subsequent brain MRI revealed five scattered, nonenhancing, white-matter lesions; including a large (approximately 5 mm in diameter) lesion involving the corpus callosum and two lesions perpendicular to the left lateral ventricle. Her B12 level, HTLV-1 antibodies, vitamin E level, RPR, ANA, and Lyme titre were all within normal limits She was started on glatiramer acetate following her clinically isolated syndrome of partial transverse myelitis and MRI findings confirming dissemination in space. Two and a half years after her initial presentation, she developed right hemiparesis that resolved over a few days following treatment with intravenous steroids. She had a subsequent similar episode of left hemiparesis and hemisensory deficit two months after that, confirming a diagnosis of clinically definite multiple sclerosis.

One year after her initial presentation, she developed a sudden onset of discomfort in her legs associated with an urge to move that was worse at night and impaired sleep. The symptom resolved while walking and had been present for only 3-4 days at the time of presentation. Her last menstrual period was two weeks prior to this presentation, and she was faithfully using an oral contraceptive. Her iron level was 108 mcg/dl, iron saturation was 28.6%, and ferritin was 94 ng/ml. A brain MRI did not reveal any new lesions. Her symptoms resolved a few days after a course of intravenous methylprednisolone (1 g daily for three days), and she had no recurrence over the next three years.

## 2. Discussion

Restless legs syndrome is a movement disorder characterized by distressing urge to move the legs (akathisia) associated with discomfort and that is brought on by rest, relieved by movement or walking, and become worse at night or in the evening [1]. The patient described above fulfilled all of these diagnostic criteria. Her rather abrupt onset suggested the possibility of restless legs syndrome secondary to an underlying cause, such as pregnancy or iron deficiency. However, her diagnostic evaluation excluded these possibilities, and her clinical course and probable response to steroids suggested that her restless legs syndrome was due to an acute exacerbation of her multiple sclerosis.

Several studies have reported an increased incidence of restless legs syndrome in patients with multiple sclerosis, have suggested both a role for restless legs syndrome in MS-related fatigue, and have suggested that multiple sclerosis is a cause of secondary restless legs syndrome [2–5]. However, none of these studies describe self-limited restless legs syndrome occurring as an acute exacerbation of multiple sclerosis as in this case. Manconi et al. demonstrated a correlation between RLS and cervical cord lesion burden [6]. This patient had a known cervical cord plaque, and this correlation might also explain why no new intracranial lesion was detected during her acute presentation with RLS. 

Restless legs syndrome is commonly idiopathic, but it has been associated with a number of conditions other than multiple sclerosis, including pregnancy, renal failure, peripheral neuropathy, and iron deficiency [1, 7]. Iron deficiency has been recognized in several studies as a cause for secondary restless legs syndrome, particularly at serum ferritin concentrations of less than 50 ng/ml [7–10]. The pathophysiology of even idiopathic restless legs syndrome is probably related to low brain iron levels [1]. MRI, CSF, and neuropathological studies have all implicated central nervous system iron deficiency in the pathophysiology of restless legs syndrome, even in the absence of systemic iron deficiency [11–16]. Recently, there has been interest in the role of iron in MS and iron deposition because cerebral venous insufficiency has been suggested as a proposed mechanism for the disease [17]. Haacke et al. reported MRI evidence of iron accumulation in the basal ganglia and thalamus of MS patients [18]. However, CSF studies have demonstrated normal CSF ferritin levels in MS [19]. Furthermore, iron appears to be an important cofactor in CNS myelination, and van Toorn et al. have reported two cases of iron deficiency associated with tumefactive demyelination in children [20–22].

The pathophysiology of MS consists of both an inflammatory demyelinating component and an axonal degeneration component [23]. LeVine et al. reported normal CSF ferritin, transferrin, and iron concentrations in MS patients with stable or relapsing and remitting MS, but elevated levels in the CSF of patients with chronic progressive MS [24]. Therefore, it is likely that the pathophysiology of RLS in MS patients is related to the inflammatory, demyelinating component of the disease which may be associated with low or normal CNS iron levels, rather than due to axonal degeneration, which is associated with CNS iron accumulation. The observation that RLS is associated with spinal cord plaques would also suggest a relationship to the inflammatory and demyelinating component of disease [6]. This patient, presenting with transient RLS due to an acute, inflammatory exacerbation of her multiple sclerosis, further supports the notion that the pathophysiology of RLS in multiple sclerosis is related to autoimmune inflammatory demyelination.
